# Changes in elastin, elastin binding protein and versican in alveoli in chronic obstructive pulmonary disease

**DOI:** 10.1186/1465-9921-9-41

**Published:** 2008-05-18

**Authors:** Mervyn J Merrilees, Pamela ST Ching, Brent Beaumont, Aleksander Hinek, Thomas N Wight, Peter N Black

**Affiliations:** 1 Department of Anatomy with Radiology, Faculty of Medical and Health Sciences, The University of Auckland, Auckland, New Zealand; 2 Cardiovascular Research Program, Hospital for Sick Children, Toronto, Canada; 3 Hope Heart Program at Benaroya Research Institute, Virginia Mason, Seattle, WA, USA; 4 Department of Pharmacology & Clinical Pharmacology, Faculty of Medical and Health Sciences, The University of Auckland, Auckland, New Zealand

## Abstract

**Background:**

COPD is characterised by loss of alveolar elastic fibers and by lack of effective repair. Elastic fibers are assembled at cell surfaces by elastin binding protein (EBP), a molecular chaperone whose function can be reversibility inhibited by chondroitin sulphate of matrix proteoglycans such as versican. This study aimed to determine if alveoli of patients with mild to moderate COPD contained increased amounts of versican and a corresponding decrease in EBP, and if these changes were correlated with decreases in elastin and FEV_1_.

**Methods:**

Lung samples were obtained from 26 control (FEV_1 _≥ 80% predicted, FEV_1_/VC >0.7) and 17 COPD patients (FEV_1 _≥ 40% – <80% predicted, FEV_1_/VC ≤ 0.7) who had undergone a lobectomy for bronchial carcinoma. Samples were processed for histological and immuno-staining. Volume fractions (*V*_v_) of elastin in alveolar walls and alveolar rims were determined by point counting, and versican and EBP assessed by grading of staining intensities.

**Results:**

Elastin *V*v was positively correlated with FEV_1 _for both the alveolar walls (r = 0.66, p < 0.001) and rims (r = 0.41, p < 0.01). Versican was negatively correlated with FEV_1 _in both regions (r = 0.30 and 0.32 respectively, p < 0.05), with the highest staining intensities found in patients with the lowest values for FEV_1_. Conversely, staining intensities for EBP in alveolar walls and rims and were positively correlated with FEV_1 _(r = 0.43 and 0.46, p < 0.01).

**Conclusion:**

Patients with mild to moderate COPD show progressively increased immuno-staining for versican and correspondingly decreased immuno-staining for EBP, with decreasing values of FEV_1_. These findings may explain the lack of repair of elastic fibers in the lungs of patients with moderate COPD. Removal of versican may offer a strategy for effective repair.

## Background

Chronic obstructive pulmonary disease (COPD) is characterised by loss of elastin in the alveolar walls [1-3]. Recently, we reported that in patients with mild to moderate COPD (GOLD stages 1 and 2) the decreased elastic fiber content of alveoli and small airways strongly correlates with decreased FEV_1 _[4], consistent with a central role for the loss of elastin in the airflow obstruction in COPD.

In COPD, elevated levels of the elastin degrading proteinases MMP-2 and MMP-9 have been found in lung tissue [5,6], alveolar macrophages [7], and sputum of subjects with COPD [8,9] and may contribute to the loss of elastic fibers in COPD. There is evidence from animal studies that repair processes are also activated in the parenchyma of lungs affected by emphysema, and that synthesis of extracellular components, including elastin and collagen, is increased. The elastin, however, is abnormally organised and distributed, and not laid down in functional networks of fibers [10-12].

Recent studies on the assembly of elastic fibers point to an inhibitory role for the matrix proteoglycans. Assembly of elastic fibers occurs in the extracellular matrix immediately adjacent to the cell surface and is mediated by the 67 kD cell surface receptor elastin binding protein (EBP). EBP, an inactive splice variant of β galactosidase, chaparones tropoelastin through the Golgi and endosomal compartments of elastogenic cells to the cell surface where it delivers tropoelastin to growing fibers [13]. This process is inhibited by a high concentration of pericellular galactosugars, such as the chondroitin sulphate (CS) chains of the proteoglycans versican and biglycan. Chondroitin sulphate chains bind to EBP and cause premature shedding of EBP from the cell surface. As a result, tropoelastin is not presented to the microfibrillar scaffolds and fiber formation is impaired [14,15].

Versican-mediated inhibition of elastic fiber assembly may also occur in lung tissues. In lymphangioleiomyomatosis (LAM), thickened alveolar walls and expanded interstitial tissues stain strongly for versican, as well as biglycan. These proteoglycan-rich regions are notably devoid of elastic fibers and stain weakly for EBP [16]. Total elastin content of LAM lungs, however, is not reduced and interstitial regions that have a low content of proteoglycan stain strongly for EBP and contain bundles of elastic fibers. These fibers, however, are not arranged in a network, indicating a problem with functional architecture rather than impairment of synthesis.

These findings raise the possibility that elastic fiber repair processes in COPD may be inhibited by an increased content of matrix proteoglycans. In this study, we asked the questions, do the lungs of patients with mild to moderate COPD stain more intensely for versican and less intensely for EBP, and does FEV_1 _predict those changes?

## Materials and methods

The lung tissue used in this study has been described previously [4]. The study was conducted using archived formalin fixed, paraffin embedded tissues from patients who had one or more lobes resected for bronchial carcinoma. The specimens were identified using the computerized records of the Department of Pathology, Green Lane Hospital. The operations were performed between January 1992 and September 1996. Further information, including smoking history, past medical history, medication and preoperative lung function, were obtained from the patient's hospital notes. The patients were classified as control subjects or COPD on the basis of their lung function. The control subjects had FEV_1 _≥ 80% predicted and FEV_1/_FVC ≥ 0.7. Patients with FEV_1 _<80% and FEV1/FVC ≤ 0.7 were classified as COPD. (For characteristics of subjects who provided archival tissue see Table 1, reference 4.) Patients with a diagnosis of asthma, bronchiectasis or interstitial lung disease were excluded and there were no changes seen in the tissue sections from the included subjects to suggest these diagnoses. Samples were obtained from 26 control and 17 COPD subjects ranging in age from 58 to 90 and 61 to 84 years respectively. Approval was obtained from the Auckland Ethics Committee to conduct the study.

All samples had been fixed in neutral buffered 10% formalin and embedded in paraffin. Histological- and immuno-staining was performed on 4 μm sections mounted on glass slides. Elastic fibers were visualised by elastin van Gieson staining as previously described [4], and versican, and elastin binding protein (EBP) by immuno-staining with monospecific polyclonal antibodies raised in rabbits. Proteoglycan antibodies (versican LF99, biglycan LF121 and decorin LF122) were kindly provided by Dr Larry Fisher, Craniofacial and Skeletal Disease Branch, National Institutes of Dental Research, NIH, Bethesda, Maryland. Anti- EBP antibody, raised to the synthetic peptide reflecting the elastin binding domain of the spliced variant of human β-galactosidase was previously described by Hinek and colleagues [17].

Deparaffinised sections were hydrated in tris buffered saline (TBS) for 3 × 5 mins, incubated with 0.03% peroxidase block for 10 mins, washed in TBS 3 × 5 mins, and incubated with primary antibodies (versican, biglycan and decorin at 1:1000, and EBP at 1:500) for 1 hr and 1.5 hr respectively. Following primary incubation, sections were washed 3 × 5 mins in TBS, incubated for 30 mins in secondary HRP rabbit polymer, washed 3 × 5 mins in TBS and stained with DAB chromogen for 6 mins. Sections were then washed twice in distilled water, counterstained with Harris' Haematoxylin and differentiated in tap water for one minute, dehydrated and mounted in hystomount. Control sections for immuno-staining received identical treatment, except for incubation for 1 hr in TBS in place of the primary antibodies.

The volume fraction % (*V*v) of elastic fibers was determined as a percentage of total tissue volume by point counting using a 100 point grid as described previously [4]. For each lung sample, 10 random sites were sampled and mean elastin *V*v determined for alveolar wall and alveolar rim regions separately. The analysis was performed by an investigator (PC) who was blinded to the patient's lung function. The sections were examined under a light microscope at 40× magnification linked by a video camera to a computer screen. The on-screen magnification was 400×. A 100 point grid (covering 2,500 sq microns) was overlaid on the computer screen and the volume fraction percent calculated from the number of times a darkly stained elastic fiber registered as a hit (i.e. fell on the grid). This was expressed as a percentage of the total number of times that alveoli walls, alveolar rims registered as a hit on the grid. For each patient 10 sites were randomly sampled for alveoli and for alveolar rims. For each patient the mean number of alveolar tissue points sampled was 420 ± 118 (SD) and for alveolar rim regions 520 ± 98 (SD).

Immuno-staining intensities were scored on a scale of 0 (no staining) to 5.0 (intense) under a light microscope (Olympus U-PMTVC) with half grades (eg 3.5) assigned if there was indecision in applying a whole integer. Generally, staining intensities for EBP were less than for versican, but with the most intense assigned a value of 5 so that the range in intensities could be fully assessed. Assessment of intensities was carried out under a 20× objective lens with a minimum of 10 random sites sampled for each patient. The scorer (PC) was blinded to the patient codes. For each lung sample, the alveolar walls and alveolar rim regions were scored separately. To validate the grading system, versican scores for all subjects were collected from two independent observers (PC and BB), both blinded to the patient codes, and the scores compared using Spearman's rank correlation coefficient test. The concordance between the observers was high with r_S _= 0.857 (p < 0.001).

Data were analysed by Students T test (between groups) and by least squares linear regression (with FEV_1 _% predicted, FEV_1 _/VC or elastin *V*v the independent variable); p of <0.05 was taken as significant.

## Results

### Elastin staining

**Figure 1:**
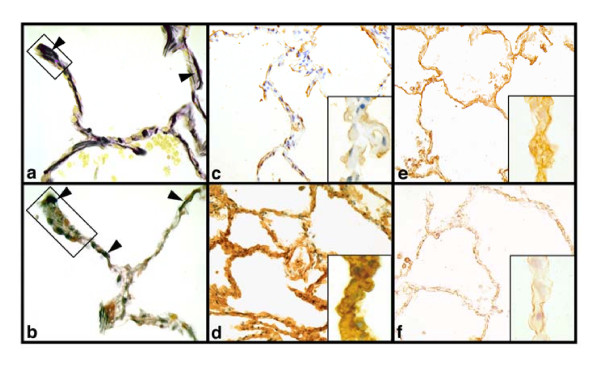
**Lung sections stained for elastin, versican and elastin binding protein**. Control (upper panels) and COPD (lower panels) lung sections stained for elastin (**a,b**)(x250), versican (**c,d**) (x100) and elastin binding protein (EBP) (**e,f**) (x100). Arrow heads indicate elastin and the boxes in **a **and **b **indicate alveolar rim regions. Staining patterns demonstrate the reciprocal relationships between versican and elastin and EBP. Inserts show segments of alveolar wall (× 500). Staining intensity values are 1 for **c **and **f **and 5 for **d **and **e**.

Elastin van Gieson staining (Fig 1a,b) showed reduced elastic fiber staining in the both alveolar wall and alveolar rim regions of COPD lungs compared with control lungs. As reported previously [4] the mean alveolar wall elastin *V*_v _in COPD lungs (18.6% ± SD 5.5) was significantly lower (p < 0.001) than in control lungs (32.8% ± 7.6). The elastin content of the alveolar rims, which was higher than in the alveolar walls, was also reduced (Figure 1a,b), but to a lesser extent. The mean elastin *V*_v _in the COPD group (31.5% ± 6.3) was significantly lower (p < 0.002) than in controls (39.0% ± 7.9). The higher *V*v of elastin in the alveolar rims of controls, compared with the alveolar walls of controls, is consistent with the visual concentration of circumferentially arranged elastic fibers around the rims of alveoli.

**Figure 2:**
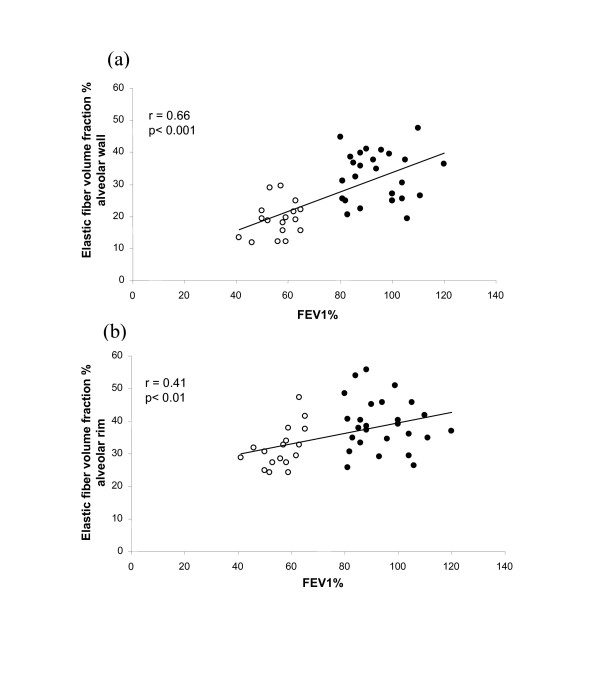
**Relationship between elastin and FEV_1_**. Relationship between elastic fiber volume fraction % of alveolar wall (a) and alveolar rims (b) and FEV_1_% in control (closed circles) and COPD (open circles) patients. (Data for (a) from [4] with permission.)

In our previous study [4] we also found that a decrease in FEV_1 _was accompanied by a decrease in elastin *V*v (Figure 2a,) for alveolar wall (r = 0.66, p < 0.001). We report here a similar relationship between FEV_1 _and *V*v of elastin in alveolar rims (Figure 2b) (r = <0.41, p < 0.01). Similar correlations were found for FEV_1_/VC and elastin *V*v (data not shown).

### Versican staining

**Figure 3:**
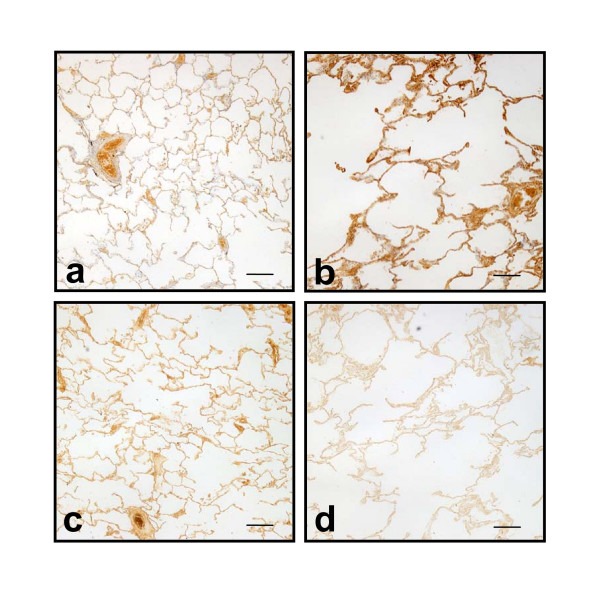
**Reciprocal relationship between versican and elastin binding protein**. Low power images of lung sections from a control patient (**a,c**) and a COPD patient (**b,d**) immunostained (using DAB chromogen) for versican and EBP. For each patient the images were taken from the same regions. Staining patterns show the reciprocal relationship between versican and EBP and the COPD lung shows the typical emphysematous structure. Scale bars = 400 μm

COPD lung samples stained more strongly for versican than control lung samples (Figure 1c,d). Mean staining intensity scores for alveolar wall of COPD samples were significantly (p < 0.002) higher than control samples (3.5 ± SD 1.5 vs 2.1 ± 1.3), as were scores for the alveolar rim regions (3.6 ± 1.5 vs 2.1 ± 1.3; p < 0.001). The stronger staining for versican and the emphysematous nature of COPD lung compared with control lung was evident on low power images of lung parenchyma (Figure 3a,b).

**Figure 4:**
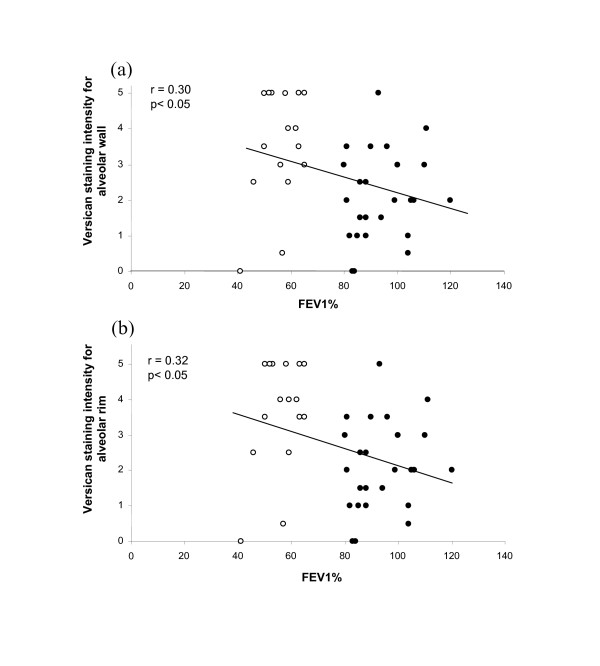
**Relationship between versican and FEV_1_**. Relationship between versican immuno-staining intensities for alveolar wall (a) and alveolar rims (b) and FEV_1_% in control (closed circles) and COPD (open circles) patients.

**Figure 5:**
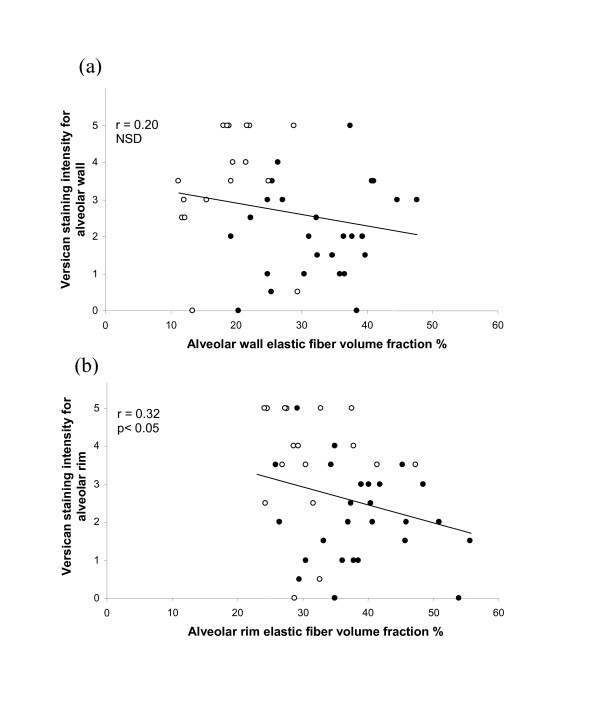
**Relationship between versican and elastin**. Relationship between versican immuno-staining intensities for alveolar wall (a) and alveolar rims (b) and elastic fiber volume fraction % in control (closed circles) and COPD (open circles) patients.

Versican staining intensities for both alveolar wall and alveolar rim were significantly and negatively correlated with FEV_1 _(r = 0.30 and r = 0.32 respectively, p < 0.05) (Fig 4a,b), with the highest staining intensities occurring in COPD patients with FEV_1 _less than 60% of predicted. Similarly, versican intensity was negatively correlated with elastin *V*v in alveolar rim regions (r = 0.32, p < 0.05) (Figure 5b). For alveolar wall this correlation was again negative but did not reach significance (r = 0.20) (Figure 5a).

### EBP staining

**Figure 6:**
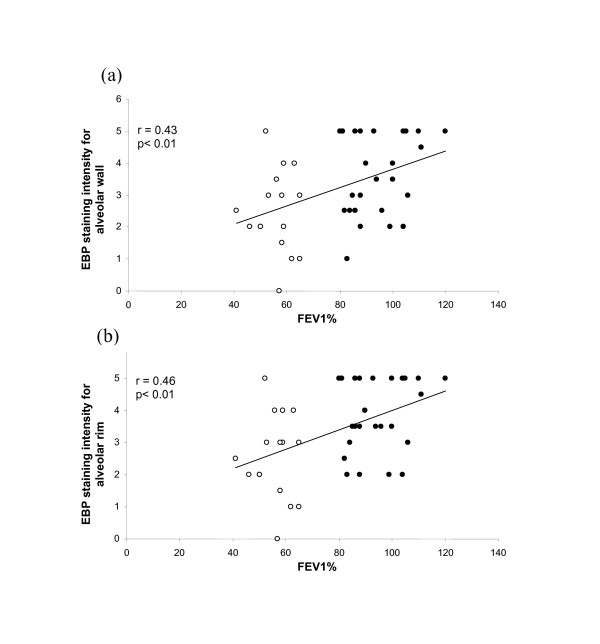
**Relationship between elastin binding protein and FEV_1_**. Relationship between EBP immuno-staining intensities for alveolar wall (a) and alveolar rims (b) and FEV_1_% in control (closed circles) and COPD (open circles) patients.

**Figure 7:**
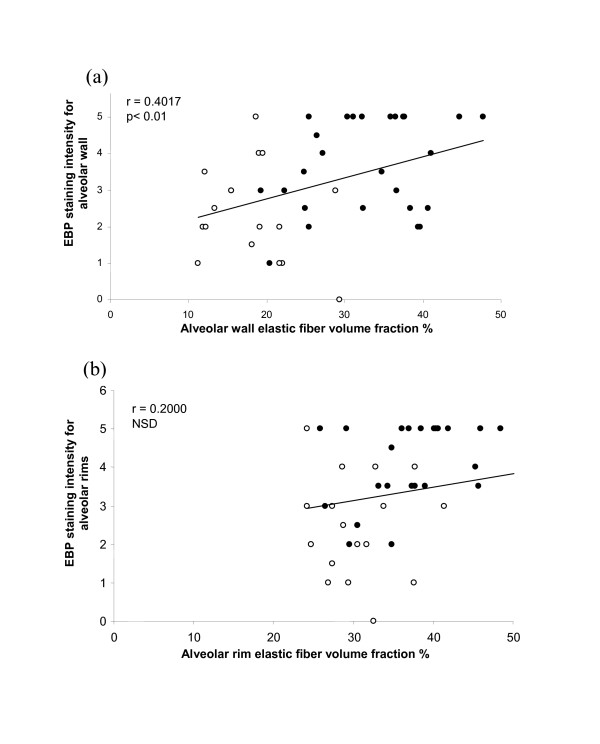
**Relationship between elastin binding protein and elastin**. Relationship between EBP immuno-staining intensities for alveolar wall (a) and alveolar rims (b) and elastic fiber volume fraction % in control (closed circles) and COPD (open circles) patients.

Immuno-staining of alveolar walls with anti-EBP antibody was significantly (p < 0.003) more intense in control lungs (mean 3.7 ± 1.3) than in COPD lungs (mean 2.4 ± 1.3). EBP intensity in the alveolar rims of controls (3.9 ± 1.1) was similarly higher (p < 0.001) than in COPD patients (2.8 ± 1.3). As for elastin volume fractions, EBP showed a reciprocal pattern to versican; intensities for both regions were significantly (p < 0.01) and positively correlated with FEV_1 _(r = 0.43 alveolar wall, r = 0.46 for rims) (Figure 6a,b). This reciprocal relationship was evident in low power images of control and COPD lung (Figure 3c,d) as well as in higher power images (Figure 1e,f). EBP similarly showed a positive relationship with elastin *V*v for alveolar wall (r = 0.40, p < 0.01), and a non-significant trend for the alveolar rims (Figure 7a,b).

Immuno-staining was also undertaken for two other matrix proteoglycans, biglycan and decorin. Although present at low levels in the walls and rims of alveoli, staining intensities were similar in both control (biglycan 1.0 ± 0.6, decorin 2.0 ± 0.8) and COPD patients (biglycan 1.2 ± 0.9, decorin 2.1 ± 1.3) and neither proteoglycan showed any relationship with FEV_1 _or with elastin *V*v.

## Discussion

A reciprocal relationship between versican and elastin and EBP has been previously demonstrated for vascular [18] and dermal tissues [19], and also for the rare lung condition lymphangioleiomyomatosis [16]. The findings of this present study show that the same relationship occurs in lung parenchyma of patients with mild to moderate COPD, and may explain, at least in part, why restoration of a functional elastic fiber network does not occur in lungs affected by COPD, despite a demonstrated capacity of emphysematous lung tissue to re-synthesise elastin and other matrix components.

In proposing a functional relationship between versican content and deposition of elastic fibers it is recognised that the immuno-staining approach used here does have limitations. For the samples available it was not possible to determine protein levels for versican and EBP and an assumption is made that staining intensities reflect content. Unmasking of epitopes with progression of COPD, however, could result in increased staining intensities without an increase in versican, although other studies, for example on human vessels, have shown a good correlation between immuno-staining intensities, precursor incorporation, and extracted amounts of proteoglycan [20,21]. Additionally, the versican antibody recognises all forms of versican, including the chainless variant V3, and it could be possible that V3 is upregulated in COPD. Other studies however, indicate that this is highly unlikely as V3, when present, is expressed at low levels [22], and attempts to understand possible functions have been through overexpression studies [18,19,35]. Further, we also recognise that our method of quantifying the amount of elastin does not provide a measure of the quality of the elastic fibers. The confocal images, however, indicate significant changes to the elastic fiber network that likely affect alveolar function.

Several experimental studies, reviewed by Snider et al [10], and by Chambers and Laurent [12] have reported that matrix proteins, including elastin and collagen, are re-synthesised in emphysematous lungs in animal models but that deposition of these components does not result in restoration of physiological function. Morris et al [23] showed that an elastase insult to hamster lungs resulted in new deposits of elastin close to cell surfaces, but a failure to form fibers. Osman et al [24] similarly demonstrated re-synthesis of elastin through measurement of incorporated ^14^C into desmosine and isodesmosine in a hamster model and additionally showed that re-synthesis was decreased by cigarette smoke which inhibited lysyl oxidase activity. Similarly, investigations on human emphysematous lungs have reported re-synthesis of matrix proteins, but with changed distribution patterns of collagens and elastin. An early study by Belton et al [25] and a later study by Fukuda et al [26] suggested that new elastin deposits may have been formed but that they were abnormal and included clumping of fibers in the free edges or rims of alveolar walls.

Recent evidence points to versican as an important inhibitor of elastic fiber formation and our findings of increased versican in COPD and decreased EBP provide for a mechanism. It has been previously established that tropoelastin present in endosomal and Golgi compartments is escorted by the 67-kDa EBP which protects this non-glycosylated and hydrophobic precursor of elastin from premature intracellular self-aggregation and association with serine proteinases [27]. Of particular importance was the observation that 67-kDa EBP also has a separate galactolectin domain and that binding of galactosugars to this site induces conformational changes in the EBP molecule, resulting in its dissociation from tropoelastin [28,29,14]. This led to the paradigm, that the release of newly secreted tropoelastin molecules from their EBP transporters, is highly coordinated and occurs on the cell surface, following the interaction between EBP and galactosugar moieties, presumably those protruding from carbohydrate chains of glycoproteins forming the microfibrillar scaffold of new elastic fibers [30,31].

On the other hand, it has been also established that such coordinated assembly of tropoelastin into elastic fibers can be disrupted by pericellular accumulation of galactosugar-bearing moieties, such as chondroitin sulfate or dermatan sulfate, which induce premature shedding of the EBP from the cell surface and release of tropoelastin, away from microfibrilar acceptors [14,17,32-34]. Thus, high levels of versican in the pericellular coat could inhibit fiber assembly even though there may be adequate synthesis of both tropoelastin and EBP. Interestingly, under normal circumstances, the efficiency of elastogenesis does not generally exceed 40–60% (unpublished data of AH) with proportionately more tropoelastin than EBP. The recycling of EBP likely mitigates against this imbalance, but displacement of EBP by versican would affect recycling. We do not know the minimal concentration of versican required to completely inhibit elastic fiber assembly.

Evidence suggests that impaired elastic fiber assembly, caused by pericellular accumulation of galactosugar-containing moieties could be reversible. Recently, it has been demonstrated, for vascular smooth muscle cells, that tropoelastin synthesis and elastic fiber assembly can be enhanced by reducing the amount of versican in the cell coat. This has been achieved by two methods, by overexpressing the glycosaminoglycan deficient variant of versican, V3 [35], which is believed to displace the larger versican variant V1 from its attachment to hyaluronan, and by reducing all versican variants at source by overexpressing a versican antisense sequence [18]. *In vivo*, neointima formed from smooth muscle cells overexpressing either V3 or versican antisense, is enriched in elastin. Significantly this elastin is organised as parallel arrays of circumferentially arranged fibers and lamellae that resemble those in developing media, indicating that a functional arrangement can be achieved *in vivo *by manipulating levels of versican. In this model, the level of EBP in the neointima was reciprocally related to the level of versican, as found for this present study of COPD lungs. Overexpression of V3, leading to decreased cell-associated chondroitin sulphate, has also been used successfully to restore elastic fiber formation in cultures of skin fibroblasts from Costello Syndrome patients who have a deficiency of elastic fibers due to over production of CS proteoglycans [19].

There are reports of increased glycosaminoglycan content in emphysematous lungs [36,37] although with dermatan rather than chondroitin sulphate being increased. In severe emphysema, the interstitial proteoglycans decorin and biglycan are reported to be decreased [38] but this was not found in this present study which examined patients with much milder emphysema. Increased versican has been reported for various lung diseases including fibrosis [39], granulomatous diseases [40], asthma [41,42], and LAM [16]. In the latter the total amount of elastin was increased, consistent with re-synthesis, but the fibers were often aggregated into bundles between elastin-free myxoid regions rich in versican and biglycan. In a recent study it has been demonstrated in developing lung that versican levels are initially raised and correlated with high tissue volume, and as development and alveolarization proceeds the levels of versican decrease, accompanied by a decrease in hydrated tissue volume. Significantly this decrease in versican precedes the rise in elastin content [43].

The findings from this present study raise a testable hypothesis, namely that repair of the elastin network in lung parenchyma, and restoration of physiological function, might be achievable, at least in mild COPD, by reducing the versican content of alveolar wall. Lowering of versican in such lungs might be particularly effective as alveoli would still be subjected to the mechanical movements of ventilation that likely have a role in determining the pattern and placement of elastic fibers during repair. The elastic fibers in the alveolar rims may be particularly important. These fibers are thicker and more prominent than those forming the elastic fiber network in the alveolar wall [44] and are important developmentally in defining the septa between alveoli [45]. Restoration of fibers in this region may be crucial to lung function. Moreover, more efficient assembly of the secreted tropoelastin may lead to a decrease in the level of free tropoelastin-derived peptides that are capable of inducing production of elastolytic MMP's [46]. Notably, in our study, the loss of elastin in the alveolar rims with decreasing FEV1 was less than for alveolar wall (Fig 2), possibly indicative of repair and maintenance despite degradative processes. Interestingly, the synthesis of elastin is greater in this region after an elastase challenge [47].

The loss of alveolar structure in more severe COPD clearly presents a much greater challenge for effective remodelling and the argument for proteoglycan-modulated repair that is proposed here may only apply to mild COPD where the architecture of the lung is not greatly changed.

## Competing interests

The authors declare that they have no competing interests.

## Authors' contributions

MJM conceived and helped design the study, supervised the data collection and analysis, and drafted the manuscript. PSTC was responsible for collecting and analysing the morphometric data, for carrying out the immuno-staining, and for preparing the figures. BB supervised the morphometric analyses, developed the immuno-staining techniques, and helped prepare the final figures. AH raised and characterised the EBP antibody, helped interpret the findings, and assisted in drafting the manuscript. TNW contributed to the design of the study, to critical analysis of the data, and helped draft the manuscript. PNB was involved in the conception and design of the study, sourced the lung tissue, provided the clinical expertise for interpretation of the data, and helped draft the manuscript. All authors read and approved the final manuscript.
